# Differential Roles of the PKC Novel Isoforms, PKCδ and PKCε, in Mouse and Human Platelets

**DOI:** 10.1371/journal.pone.0003793

**Published:** 2008-11-24

**Authors:** Catherine J. Pears, Kelly Thornber, Jocelyn M. Auger, Craig E. Hughes, Beata Grygielska, Majd B. Protty, Andrew C. Pearce, Steve P. Watson

**Affiliations:** 1 Department of Biochemistry, University of Oxford, Oxford, United Kingdom; 2 Centre for Cardiovascular Sciences, Institute for Biomedical Research, Division of Medical Sciences, The Medical School, University of Birmingham, Birmingham, United Kingdom; Illinois Institute of Technology, United States of America

## Abstract

**Background:**

Increasing evidence suggests that individual isoforms of protein kinase C (PKC) play distinct roles in regulating platelet activation.

**Methodology/Principal Findings:**

In this study, we focus on the role of two novel PKC isoforms, PKCδ and PKCε, in both mouse and human platelets. PKCδ is robustly expressed in human platelets and undergoes transient tyrosine phosphorylation upon stimulation by thrombin or the collagen receptor, GPVI, which becomes sustained in the presence of the pan-PKC inhibitor, Ro 31-8220. In mouse platelets, however, PKCδ undergoes sustained tyrosine phosphorylation upon activation. In contrast the related isoform, PKCε, is expressed at high levels in mouse but not human platelets. There is a marked inhibition in aggregation and dense granule secretion to low concentrations of GPVI agonists in mouse platelets lacking PKCε in contrast to a minor inhibition in response to G protein-coupled receptor agonists. This reduction is mediated by inhibition of tyrosine phosphorylation of the FcRγ-chain and downstream proteins, an effect also observed in wild-type mouse platelets in the presence of a PKC inhibitor.

**Conclusions:**

These results demonstrate a reciprocal relationship in levels of the novel PKC isoforms δ and ε in human and mouse platelets and a selective role for PKCε in signalling through GPVI.

## Introduction

The major receptors which induce platelet activation signal by different mechanisms although many converge on the activation of the protein kinase C (PKC) family of serine/threonine kinases. For example, thrombin signals through heterotrimeric Gq proteins leading to activation of phospholipase C (PLC)β isoforms, while the collagen receptor GPVI activates a distinct isoform, PLCγ2, through an immunoreceptor tyrosine-based activation motif (ITAM)-dependent pathway, involving sequential activation of Src, Syk and Tec family tyrosine kinases [1]. PLCβ and γ isoforms hydrolyse phosphatidylinositol 4,5-bisphosphate to generate the second messengers inositol 1,4,5-trisphosphate (IP_3_) and 1,2-diacylglycerol which mobilise Ca^2+^ from intracellular stores and activate classical and novel isoforms of PKC [2]. PKCs play a critical role in platelet activation as pharmacological inhibitors of PKC inhibit aggregation and secretion by the majority of agonists [3], [4]. Many PKC substrates have been identified in platelets including components of the secretory machinery and signalling molecules [5], [6], [7], [8].

PKC consists of nine structurally related isoforms, sub-divided into three groups based on mechanism of activation and structural similarities [9]. The classical PKC isoforms (α, β, γ), contain domains conferring regulation to diacylglycerol and Ca^2+^ and require both for full activation. The novel isoforms (δ, ε, θ, η) are Ca^2+^-independent whereas the atypical isoforms ι ~/λ and ζ are not directly regulated by diacylglycerol or Ca^2+^. Initial evidence for the involvement of the novel PKC isoforms in platelet activation came from studies monitoring tyrosine phosphorylation of these proteins following stimulation of human platelets. PKCδ is tyrosine phosphorylated at two distinct sites, Tyr311 and 565, in response to activation of GPVI and PAR receptors, but not by the major platelet integrin, αIIbβ3 [10], [11], [12], [13]. This provides a potential mechanism for both direct regulation of PKCδ activity and in initiating downstream signalling through recruitment of SH2 domain containing proteins. PKCθ is tyrosine phosphorylated in human platelets in response to collagen, the snake toxin Alboaggregin A (which interacts with both GPVI and GP-Ib-IX-V) and downstream of αIIbβ3 [14], [15]. PKCε has also been reported to be tyrosine phosphorylated downstream of Alboaggregin A in human platelets [11], although others have been unable to detect its expression in human platelets [16].

Further evidence for different functions of individual PKC isoforms downstream of different receptors relied on inhibitors with preferential isoform specificity *in vitro* [11], [17]. However the selectivity of these inhibitors within a cell remains unclear and many have been shown to have additional effects, including the so-called selective PKCδ inhibitor, rottlerlin [18], [19]. More recently, studies of platelets derived from mice lacking individual PKC isoforms have proven invaluable in providing information about specific roles of individual isoforms. Platelets deficient in PKCβ or PKCθ both show a deficiency in spreading on immobilised fibrinogen, consistent with a role for these isoforms in mediating signals induced by αIIbβ3 [15], [16]. In contrast, mouse platelets lacking PKCδ show enhanced aggregation and spreading when stimulated by collagen, suggesting a feedback inhibitory role [20].

The present study focuses on two novel isoforms, PKCδ and PKCε, in mouse and human platelets. Unexpectedly, we observed a reciprocal relationship in expression, with PKCδ detected at high and low levels in human and mouse platelets, respectively, and vice versa for PKCε. We reveal a novel role for PKCε in aggregation to collagen, but not to G protein-coupled receptor agonists, mediated through tyrosine phosphorylation of FcRγ-chain.

## Methods

### Reagents

Anti-PKCε (clone 21) and θ antibodies were purchased from BD Pharmingen (Oxford, UK) and anti-PKCη from Santa Cruz Biotechnology, Inc. (CA, USA). Anti-PKCδ antibodies were from BD Pharmingen (Oxford, UK) (clone 14) and Santa Cruz Biotechnology, Inc. (CA, USA) (sc-937) used for western blotting and immunoprecipitation, respectively. Polyclonal antibodies to PLCγ2 (DN84), Syk (BR15) and Btk (BL7) were kindly provided by Dr Mike Tomlinson (formerly of DNAX Research Institute, Palo Alto, CA, USA). PKCε−deficient mice [21] were bred as heterozygotes on a B6 background and all results compared to wild type litter-matched controls. P-PACK (D-Phe-Pro-Arg-chloromethylketone, HCl) and AEBSF were from Merck Biosciences Ltd (Nottingham, UK). Fibrillar horm collagen from equine tendon was purchased from Nycomed (Linz, Austria). Horm collagen is a preparation of native collagen fibrils from equine tendon which mainly contains predominantly type I collagen [22], [23]. Other reagents were from Sigma (Poole, UK), or previously described sources [24], [25], [26].

### Human and mouse platelet preparation

Studies on human platelets were carried out with ethical approval from the Central Oxford Research Committee (Ref: C00:203), with written informed consent obtained from all donors. Washed platelets were prepared from platelet rich plasma (PRP) in the presence of prostacyclin (0.1 µg/ml) [25]. Blood was resuspended in Tyrode's-Hepes buffer (134 mM NaCl, 0.34 mM Na_2_HPO_4_, 2.9 mM KCl, 12 mM NaHCO_3_, 20 mM Hepes, 5 mM glucose and 1 mM MgCl_2_, pH7.3). Animals were bred and blood removed under an approved Home Office Licence. Blood was drawn either by cardiac puncture or from the vena cavae of terminally CO_2_-narcosed mice, originally anaesthetised with gaseous isofluorane. Blood was taken into 100 µl ACD and 200 µl modified Tyrode's-Hepes buffer. Platelets were prepared as described [25], except final separation from PRP was by spinning at 1,000 g for 6 min. They were resuspended at 2–5×10^8^/ml in Tyrode's-Hepes.

### Platelet aggregation and spreading

Aggregation was performed at 37°C with constant stirring (1200 rpm) in a Chronolog 490-2D aggregometer (Labmedics, Manchester, UK) using 2×10^8^/ml platelets in a sample size of 300 µl. Secretion of ATP was measured using luciferase-luciferin reagent. Platelet spreading was carried out as described [27]. Where indicated, apyrase (2.5 units/ml) and indomethacin (10 µM) were added 60 sec before experimentation to prevent the action of released ADP or formation of thromboxane A_2_, respectively.

### Flow Cytometry

Expression of the cell surface glycoproteins was measured by flow cytometry [26].

### Immunoprecipitation and western blotting

300 µl washed platelets (5×10^8^/ml) were stimulated under stirring conditions (1200 rpm), terminated by addition of an equal volume of ice-cold 2% NP-40 lysis buffer and immunoprecipitations (IPs) carried out as previously described [28]. For PKCδ, IPs were carried out using monoclonal antibody against PKCδ from BD Pharmingen (clone 14) and resulting blots probed using anti-PKCδ antisera from Santa Cruz (sc-937). For PKCε, IPs and blots were carried out using monoclonal antibody from BD Pharmingen (clone 21). For PLCγ2, Syk and Btk IPs, proteins were sequentially immunoprecipitated from each sample, pre-clearing between each IP [25]. Densitometry was analysed using Alpha Imager 220 Documentation and Analysis System Software.

### Pleckstrin phosphorylation

Pleckstrin phosphorylation was monitored as described [29]. The levels of radioactivity in each band were measured using a Phosphorimager SI (Molecular Dynamics) and ImageQuaNT software, version 4.2a.

### Aggregate formation on collagen under shear

Aggregate formation on collagen was carried out as described [30] except that capillaries were imaged using differential interference contrast optics on a Zeiss Axiovert 200M microscope equipped with a Hamamatsu Orca 285 digital camera (Hamamatsu Photonics UK Ltd, Herts, UK). Contents of capillaries were subsequently lysed and levels of adherent platelets assessed by probing for actin [26].

### Statistical analysis of data

Unless stated otherwise, results are shown as mean±SEM. Statistical significance of differences between two means was determined by Student's unpaired T-test or one-way ANOVA. If means were shown to be significantly different, multiple comparisons were performed by the Tukey test. Probability values of *P*<0.05 were selected to be statistically significant.

## Results

### Comparison of novel PKC isoforms in human and mouse platelets

**Figure 1:**
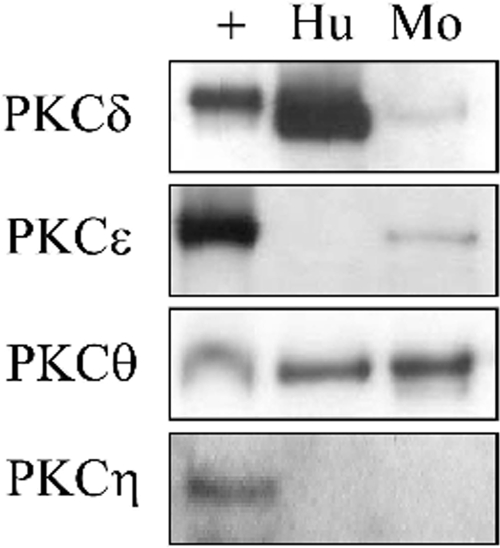
Presence of novel PKC isoforms in human and mouse platelets. Washed human (Hu) or mouse (Mo) platelets were lysed and samples separated by SDS-PAGE. The resulting membranes were blotted with antibodies raised against each isoform. Alongside each sample, the positive control (+) was run, as recommended by the antibody manufacturer. The results are representative of three experiments.

In human platelets we were able to confirm expression of PKCδ and θ, but not PKCε and η even though the antibodies detect expression of these two isoforms in other tissues and cell lines including human HeLa cells (Figure 1 and not shown). We were also unable to detect expression of PKCε in human platelets following attempts to concentrate the protein by immunoprecipitation (not shown). In contrast, mouse platelets express PKCδ, ε and θ, but not PKCη (Fig 1). Interestingly, we observed robust expression of PKCδ in human platelets and rat cerebrum using an antibody to rodent PKCδ but this isoform was hard to detect in mouse platelets. Thus, these results suggest that human platelets robustly express PKCδ but not PKCε, whereas mouse platelets express higher levels of PKCε and lower levels of PKCδ. There is precedent for protein isoforms to show species-specific expression between mouse and human platelets, with the PAR1 receptor being the principle thrombin receptor in human platelets, whereas it is absent in mouse platelets. In view of the apparent reciprocal relationship between PKCδ and PKCε, we focussed our studies on these two isoforms in mouse and human platelets.

### Investigation of tyrosine phosphorylation of PKCδ and PKCε

**Figure 2:**
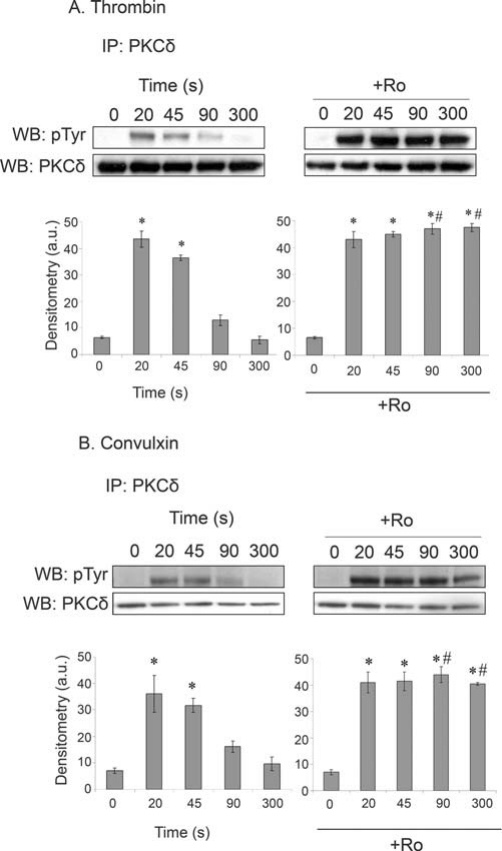
Effect of Ro 31-8220 (Ro) on PKCδ tyrosine phosphorylation. Human washed platelets in the presence of apyrase (2.5 U/ml) and indomethacin (10 µM) were treated with (A) 1 U/ml thrombin or (B) 3 µg/ml convulxin in the presence or absence of Ro 31-8220 (10 µM, 1 min) for the times shown. Immunoprecipitations and reprobes were carried out as in the methods. Histograms show mean density of bands, normalised according to the basal value. *signifies statistically significant from basal, ^#^signifies statistically significant from equivalent time point without inhibitor. Data is from 3 separate experiments.

There are several reports of robust tyrosine phosphorylation of PKCδ in human platelets, confirmed in the present study using thrombin and the two GPVI-specific agonists, convulxin and CRP. In human platelets, tyrosine phosphorylation induced by thrombin and convulxin is rapid but transient, returning to near basal levels by 90 sec. Interestingly, in both cases, tyrosine phosphorylation of PKCδ is sustained in the presence of the general PKC inhibitor, Ro 31-8220, demonstrating that it is under feedback regulation from PKC, although the responsible isoform(s) is not known (Fig 2A&B).

**Figure 3:**
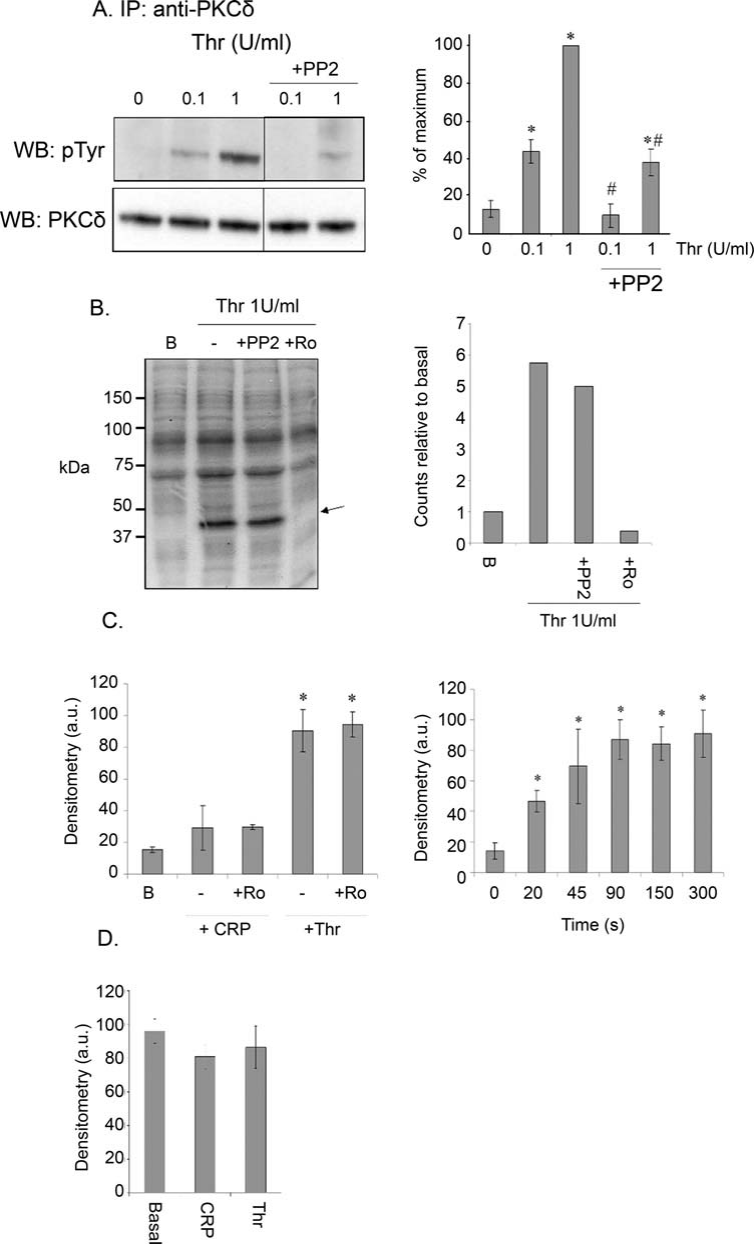
(A) Effect of PP2 on PKCδ tyrosine phosphorylation. Human washed platelets were treated with 0.1 or 1 U/ml thrombin for 1 min in the presence or absence of PP2 (10 µM, 5 min). Immunoprecipitations and reprobes were carried out as described in the methods. Histogram shows mean density of bands, normalised according to the basal value. (B) Effect of PP2 on pleckstrin phosphorylation in thrombin-stimulated platelets. Washed human platelets were labelled with radioactive ^32^P-orthophosphate and stimulated with 1 U/ml thrombin for 1 min. PP2 (10 µM, 5 min) or Ro31-8220 (10 µM, 1 min) were added where indicated. After separation by SDS-PAGE, the resulting gel was analysed using a phosphoimager to obtain radioactivity levels. Histogram shows the level of pleckstrin phosphorylation compared to basal levels. Data from one experiment, representative of two. (C) Tyrosine phosphorylation of PKCδ in mouse platelets. Washed mouse platelets were stimulated with 3 µg/ml CRP or 1 U/ml thrombin in the absence or presence of Ro31-8220 (10 µM, 1 min) (left), or with 1 U/ml thrombin for the times shown (right) in the presence of apyrase (2.5 U/ml) and indomethacin (10 µM). Immunoprecipitations and reprobes were carried out as described in the methods. Histogram shows mean density of bands, normalised according to the basal value. (D) Tyrosine phosphorylation of PKCε in mouse platelets. Washed mouse platelets were stimulated with 3 µg/ml CRP or 1 U/ml thrombin for 1 min. Immunoprecipitations and reprobes were carried out as described in the methods. All studies described in this figure were performed in the presence of apyrase (2.5 U/ml) and indomethacin (10 µM). Histograms show mean density of bands, normalised according to the basal value: *signifies values significant from basal, ^#^signifies significant from equivalent time point without inhibitor. Data is from 3 separate experiments.

To address the functional significance of tyrosine phosphorylation of PKCδ in platelets, we used the Src family kinase inhibitor PP2 to inhibit tyrosine phosphorylation of PKCδ (Fig 3A) [11], [13]. PP2 had no significant effect on phosphorylation of the major PKC substrate in platelets, pleckstrin, by a sub-maximal (0.1 unit/ml) or maximal (1 unit/ml) concentration of thrombin, although phosphorylation was completely inhibited by the pan-PKC inhibitor, Ro 31-8220 (Fig 3B and not shown). These results demonstrate either that pleckstrin is not significantly phosphorylated by PKCδ in human platelets or that tyrosine phosphorylation of PKCδ does not significantly alter its activity.

In contrast, in mouse platelets thrombin stimulated a rapid, but sustained increase in tyrosine phosphorylation of PKCδ that was unaltered in the presence of Ro 31-8220, whereas the GPVI-specific agonist CRP had no significant effect on tyrosine phosphorylation of the novel isoform either on its own or in the presence of the PKC inhibitor (Fig 3C). These results demonstrate that regulation of PKCδ phosphorylation in mouse platelets is distinct from that in human platelets. Some constitutive phosphorylation of PKCε was observed in mouse platelets, which was not altered upon stimulation by CRP or thrombin (Fig 3D), consistent with neither agonist regulating PKCε through tyrosine phosphorylation.

### Functional studies on PKCε^−/−^ mouse platelets

**Figure 4:**
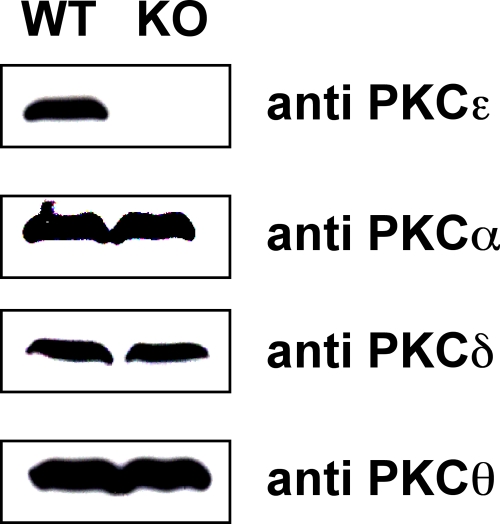
Expression of PKC isoforms in PKCε-null platelets. Platelets were prepared from PKCε-deficient mice (PKCε−/−) and wild-type littermate controls (WT) and equal protein amounts of whole cell lysates analysed by western blot, using antisera specific for individual PKC isoforms, PKC ε, α, δ and θ, as indicated.

As mouse platelets lacking PKCδ show increased aggregation in response to collagen, we investigated responses in platelets lacking PKCε. There was no significant difference in the number or size of mouse platelets in PKCε-null mice relative to controls (not shown), consistent with the report of no change in the profile of blood cells in the absence of this PKC isoform [21]. As expected, we could detect no expression of PKCε in platelets purifed from the null mice, and could find no evidence for compensatory changes in expression levels of other isoforms in platelets by western blot (Fig 4). This is consistent with previous reports of no alterations in expression of other isoforms in brain tissue from PKCε-deficient mice [21].

**Figure 5:**
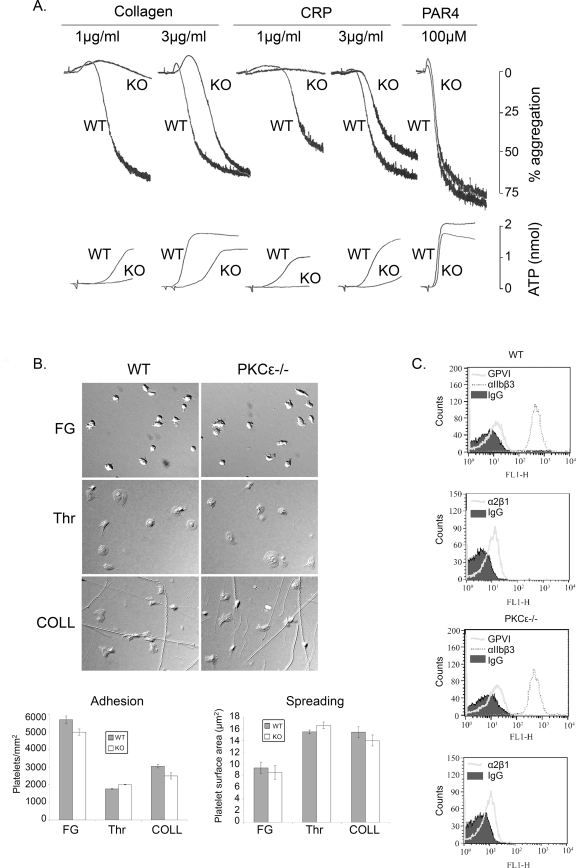
Platelet function in PKCε-null mice. (A) Aggregation and ATP secretion. Washed platelets from PKCε-deficient (PKCε^−/−^) mice or wild-type (WT) littermate controls were stimulated with the indicated concentrations of CRP, collagen or PAR4 peptide. Aggregation and dense granule secretion were investigated using light-scattering aggregometry and luciferin-luciferase luminescence, respectively. Traces are representative of between 3–7 mice. (B) Spreading. PKCε^−/−^and wild-type (WT) washed platelets in the presence of apyrase (2.5 U/ml) and indomethacin (10 µM) were exposed to surfaces of 100 µg/ml fibrinogen (FG), 1 U/ml thrombin (Thr), or 100 µg/ml collagen (COLL) for 45 min before fixing and mounting. Representative images from each condition are shown. Histograms depict levels of platelet adhesion to each surface and platelet mean surface area (spreading). The results are from one experiment that is representative of three. (C) Expression levels of surface glycoproteins. PKCε^−/−^ and WT washed platelets were incubated with FITC-labelled antisera against (upper panels) GPVI and integrin α_IIb_β_3_ and (lower panels) integrin α_2_β_1_ and expression levels analysed by flow cytometry. The shaded area represents the signal from non-specific IgG control.

Aggregation and dense granule secretion were investigated in PKCε-null platelets using light-scattering aggregometry and luciferin-luciferase luminescence, respectively. The PKCε-null platelets showed a minor delay in the onset of aggregation and a minor reduction in dense granule secretion to concentrations of thrombin (0.03 units/ml) and PAR4 peptide (100 µM) that induces near maximal aggregation in litter-matched control platelets (Fig 5A and not shown). The impairment in response was associated with a slight increase in shape change, which can be explained by the delay in onset of aggregation which has an opposing effect on light transmission. These observations suggest that PKCε makes a minor contribution, alongside other PKC isoforms, to aggregation and secretion mediated through the PAR4 receptor. Consistent with this, there was no significant change in aggregation to ADP (not shown), which induces platelet aggregation independent of PKC [31].

In contrast, there was a more marked reduction in the onset of aggregation and level of ATP secretion to low and intermediate concentrations of collagen and the GPVI-specific ligand, CRP (Fig 5A). A full recovery in aggregation, although not secretion, was seen at higher concentrations of CRP and collagen (Fig 5A and not shown). However, there was no defect in spreading of PKCε-null platelets on collagen, immobilised fibrinogen or thrombin (Fig 5B). The levels of the two collagen receptors, GPVI and α2β1, and the major platelet integrin, αIIbβ3, were similar in the PKCε-null and litter-matched control platelets (Fig 5C). These results demonstrate a selective impairment in platelet activation through the GPVI collagen receptor which is not the result of an alteration in receptor expression.

### Protein phosphorylation in PKCε^−/−^ mouse platelets

**Figure 6:**
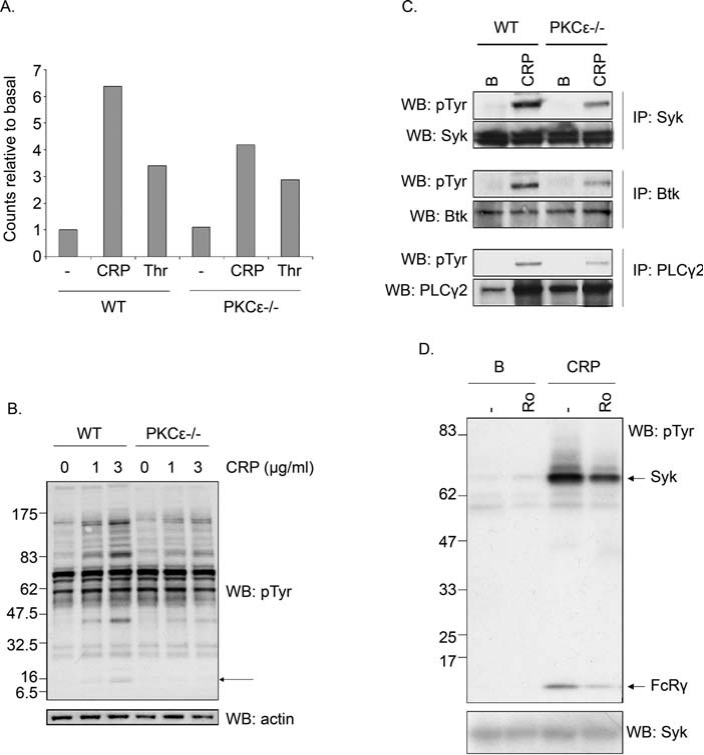
(A) Pleckstrin phosphorylation in PKCε-null platelets. Washed platelets from PKCε-deficient (PKCε^−/−^) mice or wild-type (WT) littermate controls labelled with radioactive ^32^P-orthophosphate were stimulated in the presence of apyrase (2.5 U/ml) and indomethacin (10 µM) with 0.1 U/ml thrombin or 0.3 µg/ml CRP for 1 min. After separation by SDS-PAGE the resulting gel was analysed using a phospho-imager to obtain radioactivity levels. The level of pleckstrin phosphorylation is shown in a histogram with values normalised to basal levels. B) Whole cell tyrosine phosphorylation in PKCε^−/−^ platelets. Washed platelets from PKCε^−/−^ or WT littermate controls were stimulated for 60 sec with 1 or 3 µg/ml CRP before lysis. SDS-PAGE gels were run as described in the methods. The arrow shows position of the FcR γ-chain. (C) Tyrosine phosphorylation downstream of GPVI in PKCε-null platelets. Washed PKCε^−/−^ or WT platelets were stimulated for 60 sec with 0.3 µg/ml CRP. Immunoprecipitations and reprobes were carried out as described in the methods. (D) The effect of Ro31-8220 on tyrosine phosphorylation in CRP-stimulated mouse platelets. Washed WT platelets were pre-incubated with (Ro) or without (−) Ro 31-8220 (10 µM, 1 min) prior to harvesting without (B) or with stimulation for 60 sec with 1 µg/ml CRP. Syk was immunoprecipitated and western blotted for phosphotyrosine as described in the methods. The location of Syk and the coprecipitated FcR γ-chain are shown. For all the above, results are representative of between 2–5 experiments.

The molecular basis of the reduced response was investigated by measurement of pleckstrin phosphorylation, revealing a marked reduction downstream of CRP whereas there was only a small decrease in response to thrombin in PKCε-null platelets compared to littermate controls (Fig 6A). This reduction in response to GPVI was associated with a marked decrease in tyrosine phosphorylation induced by the agonist, illustrated by measurement of phosphorylation in whole cell lysates (Fig 6B). The FcRγ-chain runs as a distinct band in the whole cell lysate and, importantly, its phosphorylation can be seen to be reduced revealing that inhibition occurs at a proximal stage in the signalling cascade. Consistent with this, immunoprecipitation studies reveal reduced tyrosine phosphorylation of several other signalling proteins that lie downstream of the FcRγ-chain in the GPVI signalling cascade, namely Syk, Btk and PLCγ2 (Fig 6C). These results therefore identify a role for PKCε in the GPVI signalling pathway that is mediated by a reduction in phosphorylation of the FcRγ-chain underlying the selective defect in response to collagen and CRP. Consistent with this, a pan PKC inhibitor, Ro 31-8220, reduces CRP-stimulated tyrosine phosphorylation in whole cell lysates (not shown) and of the FcRγ-chain and Syk in Syk immunoprecipitates in mouse platelets (Fig 6D), although interestingly a similar effect is not seen in human platelets (not shown).

### Platelet aggregation under arteriolar shear in PKCε^−/−^ mouse platelets

**Figure 7:**
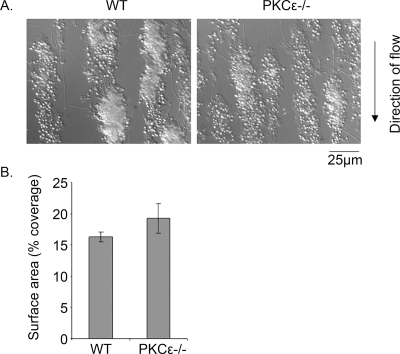
Platelet adhesion to collagen under flow in PKCε ^−/−^ mice. Blood from PKCε^−/−^ mice or wild-type (WT) littermate controls was flowed over collagen for 4 min at a shear rate of 1000 s^−1^, then rinsed with Tyrode's buffer for 5 min. A. Adherent platelets and aggregates were imaged by DIC microscope and representative images are shown. B. The surface coverage was calculated as described in the methods and is shown as the mean±s.e.m. from four experiments.

The consequence of the defect in GPVI receptor signalling on aggregate formation on a collagen surface at arteriolar shear was investigated. Blood from control and PKCε^−/−^ mice rapidly forms platelet aggregates on collagen, which continue to increase in size over the course of several minutes, with no discernable difference in either population (Fig 7A). Consistent with this, there was no significant difference in surface area coverage (Fig 7B) or the level of platelet protein as measured by western blotting for actin (not shown). The lack of an effect on platelet aggregation under arteriolar flow in the absence of PKCε is consistent with previous reports of unaltered aggregate formation on collagen under similar conditions in mice that have a reduced level of the GPVI-FcRγ-chain complex, despite the partial reduction in aggregation to collagen [26]. This is presumably because the inhibition of the response to collagen is partial and aggregate growth under arteriolar shear is driven by the secondary mediators ADP and TxA_2_.

## Discussion

The present study demonstrates a selective role for PKCε in GPVI signalling in mouse platelets with impaired aggregation and secretion to collagen and CRP. In comparison, aggregation and secretion induced through the PAR4 receptor are only marginally inhibited, consistent with a minor role for this PKC isoform in supporting activation alongside other PKC isoforms. The selective defect in GPVI signalling in PKCε-deficient platelets contrasts sharply with that of PKCδ, as platelets from mice deficient in the latter show increased filopodia formation on CRP and increased aggregation to collagen in suspension [20]. This demonstrates unique functions for the two novel PKC isoforms downstream of GPVI in mouse platelets. The defect in GPVI signalling seen in the PKCε-null mouse platelets is not due to a change in the level of expression of GPVI or α2β1. The observation that aggregation to ADP was not altered in mouse platelets argues against a role for a change in expression of integrin αIIbβ3, a result confirmed by flow cytometry.

The reduction in response to GPVI can be accounted for by a decrease in tyrosine phosphorylation at a proximal stage of its signalling cascade, as phosphorylation of the FcRγ-chain and Syk are reduced. A mechanism to explain this has been described in mast cells, where, in response to activation of the FcεRI receptor, PKC phosphorylates the FcRγ-chain on threonine-60, two residues downstream of the conserved C-terminal tyrosine residue which is phosphorylated in the ITAM, leading to Syk association [32]. Expression of a mutated form of the FcRγ-chain with threonine-60 replaced by alanine led to reduced association and activation of Syk and subsequent inhibition of degranulation in response to IgE [33]. The authors speculate that phosphorylation of the ITAM by a novel PKC isoform increases binding of Syk to the ITAM and enhances activation, illustrated by the increase in tyrosine phosphorylation of Syk and downstream proteins. We speculate that this increase in binding of Syk to the FcRγ-chain protects the ITAM moiety from dephosphorylation leading to a net increase in tyrosine phosphorylation. In mast cells, PKCδ is responsible for the phosphorylation. However, although PKCδ is expressed robustly in human platelets, it is at low levels in mouse platelets. PKCε is present in mouse platelets and, as for PKCδ, its recombinant form does mediate phosphorylation of the FcRγ-chain ITAM *in vitro* [32]. Tyrosine phosphorylation of the FcRγ-chain, Syk and downstream proteins is also reduced in the presence of a pan-PKC inhibitor in mouse platelets, confirming that the reduction in tyrosine phosphorylation of FcRγ-chain is mediated by the loss of a PKC isoform. Interestingly, a similar result is not seen in human platelets, which, although consistent with the low levels of PKCε, is surprising given the presence of PKCδ. This may reflect a further difference between mouse and human platelets in the role of novel PKC isoforms.

Our inability to detect expression of PKCε in human platelets was not expected as there are several reports describing its presence. However, Buensuceso *et al.* were also unable to detect expression of PKCε in human platelets although both positive and negative studies used the same source of antibodies as used in the present one [11], [14], [16], [17]. Our attempt to address this through concentration of PKCε by immunoprecipitation was unsuccessful. Whilst the explanation for this discrepancy is not known, and may reflect differences in batches of antibodies, the cumulative reports illustrate that PKCε is either absent in human platelets or present at a very low level.

The present study demonstrates that the tyrosine phosphorylation of PKCδ seen in human platelets is down-regulated through a PKC-dependent pathway. The molecular basis of this is not known, although it could be dependent upon the direct or indirect regulation of a protein tyrosine phosphatase. For example the SHP-1 tyrosine phosphatase interacts with PKCα and is phosphorylated on a consensus PKC phosphorylation site following human platelet activation [34]. Phosphorylation of SHP-1 negatively regulates its activity *in vitro*, but the *in vivo* consequences of phosphorylation may be more complex, with the potential to regulate substrate specificity and localisation. Interestingly, in mouse platelets tyrosine phosphorylation of PKCδ is sustained, further emphasising the species-specific functions and regulation of PKC isoforms. PAR4 has been reported to stimulate sustained tyrosine phosphorylation of PKCδ in human platelets [13], and this is the major signalling receptor for thrombin in mouse platelets. Thus, the difference in kinetics may reflect differential expression and roles of PAR1 and PAR4 in human and mouse platelets.

Although frequently used as a marker of PKCδ activation, the functional significance of its tyrosine phosphorylation is unclear. It has been reported to increase the kinase activity of PKCδ in human platelets but is not required for membrane association [12]. This study shows that blockade of Src kinases, and hence phosphorylation of PKCδ, does not alter pleckstrin phosphorylation in thrombin-stimulated platelets, arguing against direct regulation of enzymatic activity, with the caveat that PKCδ may not play a major role in regulating pleckstrin phosphorylation. The lack of Src kinase blockade on pleckstrin phosphorylation is consistent with the fact that Src kinases play a minimal role in thrombin-induced platelet aggregation [35], again questioning the significance of PKCδ tyrosine phosphoryation in this signalling pathway. The significance of tyrosine phosphorylation of PKCδ downstream of GPVI remains to be established.

In conclusion, the present study has provided further evidence for specific regulation and functions of PKC isoforms by focussing on the two novel PKCs, PKCδ and PKCε, and in particular has shown that PKCε plays a critical role in potentiating the proximal events in the GPVI signalling pathway. Further, it has also demonstrated differences in expression and phosphorylation of the two isoforms in mouse and human platelets further revealing species-specific functions of individual isoforms.
